# Electrotransfer of Different Control Plasmids Elicits Different Antitumor Effectiveness in B16.F10 Melanoma

**DOI:** 10.3390/cancers10020037

**Published:** 2018-01-29

**Authors:** Masa Bosnjak, Tanja Jesenko, Urska Kamensek, Gregor Sersa, Jaka Lavrencak, Loree Heller, Maja Cemazar

**Affiliations:** 1 Department of Experimental Oncology, Institute of Oncology Ljubljana, Zaloska 2, SI-1000 Ljubljana, Slovenia; mbosnjak@onko-i.si (M.B.); tjesenko@onko-i.si (T.J.); ukamensek@onko-i.si (U.K.); gsersa@onko-i.si (G.S.); 2 Faculty of Health Sciences, University of Ljubljana, SI-1000 Ljubljana, Slovenia; 3 Department of Cytopathology, Institute of Oncology Ljubljana, Zaloska 2, SI-1000 Ljubljana, Slovenia; jlavrencak@onko-i.si; 4 Frank Reidy Research Center of Bioelectrics, Old Dominion University, 4211 Monarch Way, Norfolk, VA 23508, USA; 5 School of Medical Diagnostic & Translational Sciences, College of Health Sciences, Old Dominion University, Norfolk, VA 23529 USA; 6 Faculty of Health Sciences, University of Primorska, Polje 42, SI-6310 Izola, Slovenia

**Keywords:** gene electrotransfer, control plasmids, murine melanoma tumor, DNA sensors, cytokines, antitumor effectiveness

## Abstract

Several studies have shown that different control plasmids may cause antitumor action in different murine tumor models after gene electrotransfer (GET). Due to the differences in GET protocols, plasmid vectors, and experimental models, the observed antitumor effects were incomparable. Therefore, the current study was conducted comparing antitumor effectiveness of three different control plasmids using the same GET parameters. We followed cytotoxicity in vitro and the antitumor effect in vivo after GET of control plasmids pControl, pENTR/U6 scr and pVAX1 in B16.F10 murine melanoma cells and tumors. Types of cell death and upregulation of selected cytosolic DNA sensors and cytokines were determined. GET of all three plasmids caused significant growth delay in melanoma tumors; nevertheless, the effect of pVAX1 was significantly greater than pControl. While DNA sensors in vivo were not upregulated significantly, cytokines IFN β and TNF α were upregulated after GET of pVAX1. In vitro, the mRNAs of some cytosolic DNA sensors were overexpressed after GET; however, with no significant difference among the three plasmids. In summary, although differences in antitumor effects were observed among control plasmids in vivo, no differences in cellular responses to plasmid GET were detected in tumor cells in vitro. Thus, the tumor microenvironment as well as some plasmid properties are most probably responsible for the antitumor effectiveness.

## 1. Introduction

Gene electrotransfer (GET) is a non-viral delivery method for transfection of cells and tissues [1,2,3]. The transfer of genetic material across the cell membrane is enabled by a transient increase in the permeability of cell membrane achieved by exposing the cells to short high voltage electric pulses. This process is referred to as electroporation (EP) [4,5]. Most commonly, plasmids; small, circular, double stranded, extra-chromosomal or episomal DNA molecules, are used for transferring the genes of interest [6,7,8]. GET is gaining in importance as delivery system in DNA vaccination and gene therapy as an alternative approach to viral vectors [9,10,11,12].

While GET is an efficient, reliable and safe in vivo transfection method, one of its shortcoming is the high cell mortality that particular pulse protocols can induce. Cell deaths after GET can be ascribed to physical damage caused by EP, such as mechanical stress, shear forces, osmotic stress, heat, and reactive oxygen species. Thus, for different applications of GET, different pulse protocols should be used and optimized for a particular application. Whereas in intratumoral GET, when targeting cancer cells, high cell mortality induced by pulse protocol and/or plasmid backbone could potentially contribute to overall effectiveness, in other GET applications such as vaccination this phenomenon is undesirable. For the purpose of DNA vaccination, the cells should survive GET and express the delivered gene of interest in order to get the desired effect [13,14,15]. Furthermore, the delivered foreign genetic material can also activate signal transduction cascades that evolved as warning systems, activated after a eukaryotic cell is exposed to the exogenous danger. For example, cytosolic DNA sensors can detect foreign DNA as pathogen-associated molecular patterns (PAMPs) or as damage-associated molecular patterns (DAMPs). Activation of these sensors mediates the activation of proinflammatory molecules that can trigger an innate and potentiate an adaptive immune response [16,17,18,19]. 

In several previous studies, we observed that intratumoral GET of different control plasmids used as controls to therapeutic plasmids produced significant but varying antitumor effectiveness, suggesting that perhaps the composition of plasmid DNA is an important variable. Increased expression of several different DNA sensors and cytokines was observed in these studies that could contribute to antitumor effect [6,17,20,21,22,23,24]. However, due to the differences in GET protocols, control plasmids and experimental tumors, the observed antitumor effects were incomparable; therefore, the current study was conducted comparing antitumor effectiveness of three different control plasmids, using the same GET parameters in B16.F10 murine melanoma tumors. We were especially interested to discover if the antitumor effectiveness could be correlated to the cytotoxic effect in vitro.

Taken together, our aim in the present study was to follow the cytotoxicity unrelated to a therapeutic transgene and antitumor effects of intratumoral GET using three different control plasmids (pControl, pENTR/U6 scr and pVAX1) in B16.F10 murine melanoma cells and tumors. Cytotoxicity and cell death mechanisms in vitro and tumor growth delay in vivo were determined. Expression of the mRNAs of selected DNA sensors and cytokines that we previously demonstrated to be overexpressed in tumor cells were quantified.

## 2. Results 

### 2.1. In Vivo Gene Electrotransfer of Control Plasmids Resulted in Significant Tumor Growth Delay and Complete Tumor Regression. These Effects Correlated with an Increased Expression of Cytokine mRNAs 

**Figure 1:**
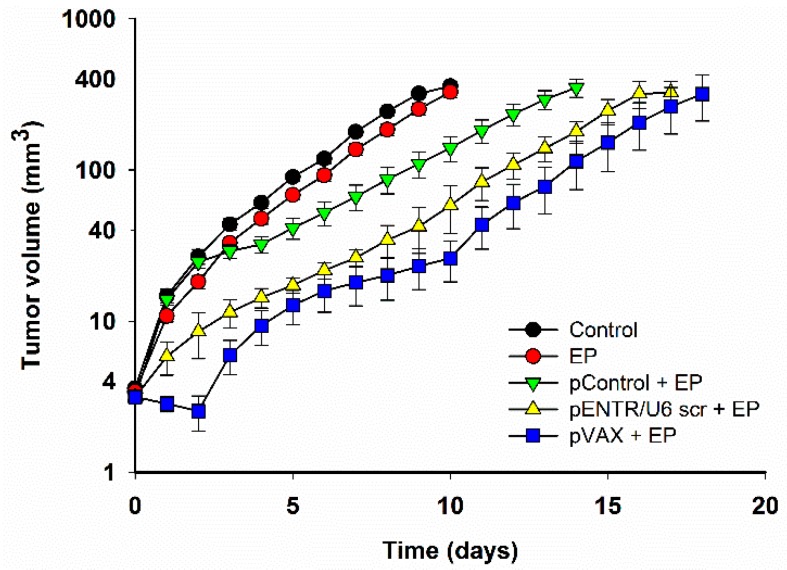
Tumor growth delay after gene electrotransfer (GET) of different control plasmids compared to control and electroporation (EP) group.

After EP of tumors alone, no significant tumor growth delay was observed (Figure 1, Table 1). Significant tumor growth delay with respect to control tumors was observed after intratumoral GET of each control plasmid. Tumor growth delay was more pronounced when pVAX1 (pVAX) was used than when pControl or pENTR/U6 scr were used in combination with the EP. 

Complete tumor regressions after GET of control plasmids were observed in all groups. Paralleling the effect on tumor growth, GET of pVAX caused the highest percent (33%) of complete responses.

**Figure 2:**
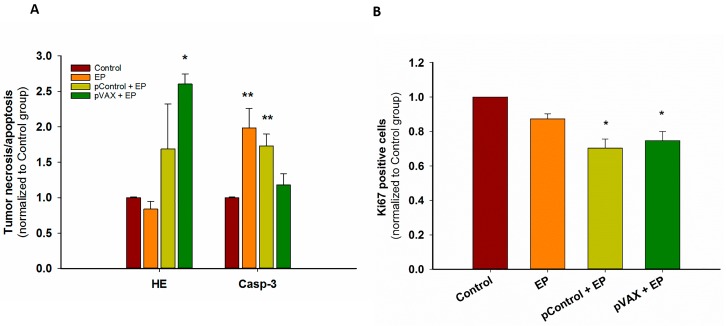
(**A**) Tumor necrosis (HE) or apoptosis (Caspase-3 positive cells (Casp-3)) after GET of control plasmids (**B**) Proliferation (Ki67 positive cells) of tumor after GET of control plasmids. * *p* < 0.05 statistically significant difference compared to control and EP group. ** *p* < 0.05 statistically significant difference compared to control.

For histological analysis of tumors, only the conditions causing a minimal (pControl) and maximal (pVAX) antitumor effect were compared to control group and EP group. First, cell death mechanisms were observed. Hematoxylin and eosin (HE) staining showed less than 20% of necrosis in control, untreated tumors, approximately 40% after GET of pControl, and approximately 60% tumor necrosis was observed after GET of pVAX plasmid. Values were normalized to control, untreated tumors and shown in Figure 2A. While GET of pVAX caused primarily necrosis, Caspase-3 staining demonstrated that EP only and GET of pControl caused primarily apoptosis (Figure 2A). The proliferation potential of the residual viable part was also assayed by Ki67 staining of tumor sections. There was a significant decrease in proliferation after GET of pVAX as well as pControl (Figure 2B). Although no statistically significant difference among two plasmids was observed, these findings confirmed our previous growth delay observations.

In our previous study, increased expression of IFN β after intratumoral GET of control plasmid gWiz Blank was observed in murine melanoma tumor in vivo, therefore we determined the expression of IFN β and TNF α after intratumoral GET of the three control plasmids examined in this current study [17]. The mRNAs of cytosolic DNA sensors DDX60, p204 and DAI/ZBP1, were also examined. Confirming our previous research, all control plasmids examined in this current study caused increased expression of IFN β in the tumors. Interestingly, TNF α was significantly increased in the tumors only in the pVAX + EP group. DNA sensor mRNAs were not statistically significantly upregulated with the exception of p204 after GET of pENTR/U6 scr (Table 2).

### 2.2. In Vitro Gene Electrotransfer of Control Plasmids Caused Necrotic Cell Death and Expression of DNA Sensor and Signaling Pathway mRNAs 

Delivery of the three plasmids were also tested in vitro to elucidate the mechanisms causing tumor growth delay and extensive tumor necrosis.

**Figure 3:**
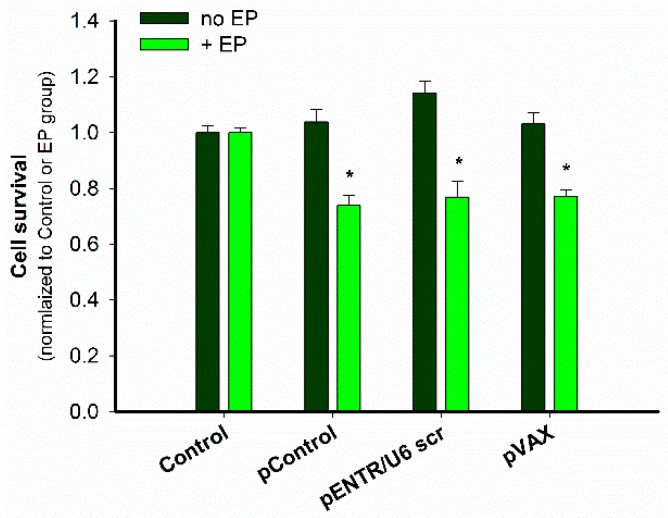
Cytotoxicity of GET of control plasmids. * *p* < 0.05 statistically significant difference compared to all control groups (no EP) and EP group.

After GET of these plasmids, a cytotoxic effect was observed in B16.F10 melanoma cells using a metabolic assay. All the plasmids were equally cytotoxic (Figure 3). 

**Figure 4:**
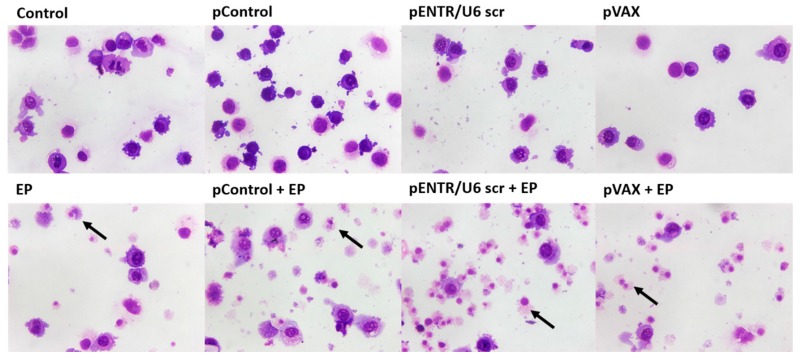
Giemsa staining showed altered cell morphology after GET of control plasmids. Black arrows indicate necrotic cells displaying fragments of cytoplasm and only an outline of the nucleus.

The cell morphology 20 h after GET of these plasmids was observed after Giemsa staining, in which non-damaged tumor cells have clearly visible violet-stained nucleus, while necrotic (damaged) cells stain preferably with eosin, resulting in lighter pink staining of the cells. Almost no cell death was observed in control samples, whereas a major increase of cell death was observed after EP alone. Fragments of dead cells were primarily observed after GET with these plasmids. The findings indicate that necrosis is the most likely cell death mechanism after GET of plasmids in vitro (Figure 4). 

**Figure 5:**
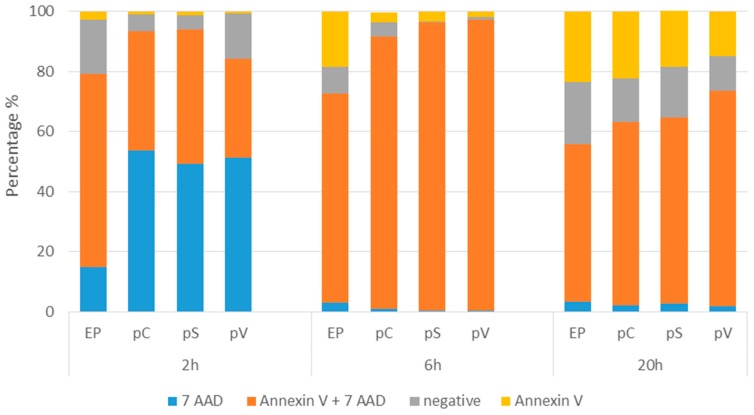
Percentage of viable cells (negative), apoptotic (Annexin V), late apoptotic/necrotic (Annexin V + 7 AAD) and necrotic (7 AAD) at 2, 6 and 20 h after GET of control plasmids. The experiment was repeated twice with three parallels.

The mechanism of this cytotoxic effect detected by decreased metabolism and changed morphology was determined by flow cytometric analysis of the cells with FITC Annexin V and 7-AAD. Only cells exposed to EP were measured since we previously observed no cell death in all other control groups (data not shown). GET of all three plasmids caused necrotic cell death as indicated by high percentages of 7-AAD-positive and Annexin V + 7-AAD-positive cells at 2, 6 and 20 h after GET (Figure 5), which confirmed the data obtained by Giemsa staining. The highest percentage of 7-AAD positive cells was measured at 2h after GET, indicating on immediate cell death caused by the EP alone or by GET, probably due to osmotic unbalance. At later time points, when the cells recovered from this immediate stress, a higher percentage of apoptotic and viable cells which started to divide were observed, specifically at 20 h (Figure 5). 

Since it was previously observed that GET of gWiz Blank plasmid induced increased expression of cytosolic DNA sensors and IFN β in B16.F10 melanoma cells, we investigated if the control plasmids analyzed in the present study had the same effect and if there was any difference among them. We previously demonstrated that the application of EP alone did not increase the expression of DNA sensors and IFN β mRNAs [17]. Cytosolic DNA sensors DDX60 and p204 (for all control plasmids) and DAI/ZBP1 (for pENTR/U6 scr + EP and pVAX + EP) mRNAs were statistically significantly increased only when compared to control group. No difference in the expression of the cytosolic DNA sensor mRNAs among the different plasmids was observed. Furthermore, the mRNA level of IFN β as well as TNF α were not statistically significantly increased comparing different plasmids with each other (Table 3). IFN β was increased only in pVAX + EP group compared to control untreated cells.

## 3. Discussion

Several previous studies have shown that different control plasmids delivered intratumorally by different GET protocols cause antitumor action in different murine tumor models, including B16.F10 mouse melanomas [17,20,21,22,25,26]. These actions include tumor growth delay, increased animal survival, complete tumor regression, and subsequent resistance to tumor cell challenge. The results of the current study, performed on B16.F10 cells and tumors with a single GET protocol but different plasmids, demonstrate that there is no difference in response to the plasmids at the in vitro level; however, the level of antitumor effect in vivo may be plasmid dependent.

After GET of three different control plasmids to murine B16.F10 melanoma tumors, significant tumor growth delay was observed. GET of the plasmid pControl had the least potent effect of the three plasmids, resulting in a low but significant growth delay (4.6 days compared to control group) and lowest rate of complete responses after treatment (1/12 mice, 8%). Conversely, after GET of pVAX, growth delay (10 days) and complete response rate (2/6 mice, 33%) were the highest of the three plasmids and statistically significantly different than after GET of pControl. In other studies, using different GET protocols pVAX also caused significant tumor growth delay and tumor regression [20]. Moreover, Marrero et al. also showed that some tumor free mice treated with GET of pVAX were immune to challenge with B16.F10 tumor cells indicating the involvement of an anti-tumor adaptive immune response [22]. This corroborated an earlier study in which approximately 70% of mice with complete tumor regression after GET of control plasmid were resistant to B16.F10 cell challenge [21]. In studies on larger tumors, the antitumor effect was also present after GET of control plasmids [17,20,22]. In B16.10 tumors, GET of the plasmid pVAX caused a higher number of complete response compared to the data obtained in our study on smaller tumors [22]. In our previous study, another control plasmid, pENTR/U6 scr, also used in this study, produced no growth delay after GET in a TS/A mammary carcinoma tumor model, which is known as poorly immunogenic or a “cold” tumor model [8,27,28,29,30,31,32]. This indicates that the effect of a control plasmid is tumor type specific and that immunologically “hot” tumors, such as B16.F10 melanomas [17] are more responsive than “cold” ones. In addition, other tumor related properties, such as metastatic potential, might contributed to different antitumor effects of control plasmids. Namely, in our previous study, pControl was used as a control plasmid in two murine melanoma models with high (B16.F10) and low (B16.F1) metastatic potential. GET of pControl produced a greater growth delay in the low metastatic type of B16 murine melanoma, B16.F1 melanoma tumors [6]. This further supports the concept that antitumor effectiveness of control plasmids is also tumor type dependent.

Previous studies indicated on activation of immune response due to the presence of plasmid DNA in the cells’ cytosol. Thus, we measured the upregulation of cytosolic DNA sensors and cytokines IFN β and TNF α. With the exception of p204, the expression of DNA sensor mRNAs in vivo was not significantly increased, which correlated with our previous findings, where only the cytokine IFN β mRNA was significantly overexpressed, comparable to this study [17]. After GET of pVAX not only IFN β, but also TNF α mRNA was increased, which is probably associated with an increased proportion of necrosis and consequently the highest tumor growth delay and complete tumor regression rate. In contrast to apoptosis, which is a programmed cell death, necrosis is an uncontrolled type of cell death. During necrosis, the cell membrane is damaged and the cell’s content is released into the extracellular environment initiating inflammation, thus inducing an intensive immune response. 

In vitro, regardless of the plasmid used, the level and type of cell death was the same for the three control plasmids. Following GET of control plasmids, the upregulation of DNA sensors was obtained, the mRNA levels of IFN β and TN F α were also increased, but not statistically significant. Binding and activation of these sensors may lead to the induction of inflammatory responses and cell necroptosis. The precise factors that influence nucleic acids binding and activation of these sensors are in general poorly characterized. DAI/ZBP1 [33,34] and DDX60 [35] bind both DNA and RNA. DNA binding by the human orthologue of p204, IFI16, is be more characterized. This protein cooperatively assembles on dsDNA greater than 60 bases pairs [36]. Therefore, although these plasmids are very similar, the structure or sequence of pVAX may better potentiate DNA sensor binding and activation. Because pENTR/U6 scr produces a small hairpin RNA, and these molecules are known to activate the RNA sensor RIG-I [37], this additional activation in cell types present in tumors that express this protein may account for the distinct response to this plasmid. Finally, approximately 15 putative cytosolic DNA sensors have been described in multiple cell types, and these plasmids could be detected by a different DNA sensor or activate a different signaling pathway in different cell types, not studied here [18,38,39]. There are several different downstream proteins, such as interleukins (IL-1, IL-12, IL-18), caspases (Casp-3, Casp-1 and Casp-8), and cyclin-dependent kinase (CDK), that could be also contribute to the observed effect [18].

The activation of cytosolic DNA sensors in vivo leads to induction of immune response. GET of control plasmids induces changes in the cytosolic DNA sensor pathway with different downstream effects, which cannot be directly ascribed to the yet known properties of the construct. The size, structure, CpG motif content or the difference in antibiotic resistance gene do not appear to play a role in the level of upregulation of cytosolic DNA sensors, since all three plasmids have similar characteristics (Table 4 Materials and methods). There are probably some other plasmid characteristics which are responsible for distinct effects of three control plasmids in the same tumor type B16.F10 as determined in the present study. In addition, the observed antitumor effects in vivo cannot be ascribed to the direct effects in tumor cells, since the effects in isolated melanoma cells after GET were the same. Therefore, other factors unrelated to tumor cells must contribute to the observed antitumor effectiveness.

The tumor microenvironment is composed of many types of cells that can contribute to the antitumor effectiveness of a particular therapy. In this study GET of pVAX induced a distinct downstream response from pControl or pENTR/U6 scr in vivo. Non-tumor cells such as tumor stroma cells, vascular cells, and particularly immune cells are likely to respond differently to GET of plasmid DNA. These cells may respond by upregulation of different DNA sensors or by no response at all. This variety of responses may muddle the results of detection assays and account for the lack of statistical significance in the in vivo studies.

In summary, although differences in antitumor effects were observed among control plasmids in vivo, no differences in cellular responses to plasmid GET were detected in tumor cells in vitro. Other cells present in the tumor, including fibroblasts, endothelial cells, and particularly immune cells may be responsible for the effects. Furthermore, the results of our study demonstrate that not only the properties of the tumor microenviroment, but also properties related to plasmids contribute to the observed antitumor effectiveness. These properties are not completely understood, therefore further studies are needed.

## 4. Materials and Methods 

### 4.1. Plasmids

Three different control plasmids, two of them pControl [6], pENTR/U6 scr [8] routinely used in our studies, and the common commercially available plasmid pVAX (pVAX1^TM^, Thermo Fisher Scientific, Waltham, MA, USA), which was specifically designed to be used for the development of DNA vaccines, were used in the experiments. Their characteristics are presented in Table 4. Amplification of the plasmids was performed in *E. coli* and plasmids purified using EndoFree Plasmid Mega Kits (Qiagen, Hilden, Germany) according to the manufacturer’s protocol. The quality and quantity of isolated plasmid DNA were determined by spectrophotometric measurements of A_260_/A_280_ ratio (Epoch Microplate Spectrophotometer, Take3™ Micro-Volume Plate, BioTek, Winooski, VT, USA) and agarose gel electrophoresis (Scie-Plas ltd, Cambridge, UK). The working concentration of 1 mg/mL (for in vitro experiments) or 4 mg/mL (for in vivo experiments) was prepared in endotoxin-free water.

### 4.2. Cells

The B16.F10 murine melanoma cell line (American Type Culture Collection, Manassas, VA, USA) was cultured in an advanced minimum essential medium (AMEM; Gibco, Thermo Fisher Scientific), supplemented with 5% fetal bovine serum (FBS; Gibco), 10 mL/l L-glutamine (GlutaMAX; Gibco), 100 U/mL penicillin (Grünenthal, Aachen, Germany) and 50 µg/mL gentamicin (Krka, Novo mesto, SIovenia) in a 5% CO_2_ humidified incubator at 37 °C. The cells were routinely tested and confirmed to be free from mycoplasma infection, using assay PlasmoTest™—Mycoplasma Detection (Invivogen, Toulouse, France) at the Institute of Microbiology and Immunology, Medical faculty, University of Ljubljana.

### 4.3. Animals and Tumors

In the experiments, 6–8 week old female C57Bl/6 mice (Envigo, Udine, Italy) were used. Mice were housed in specific pathogen-free conditions at a temperature of 20–24 °C, relative humidity 55 ± 10% and a 12 h light/dark cycle. Food and water were provided ad libitum. All procedures were approved by The Administration of the Republic of Slovenia for Food Safety, Veterinary and Plant Protection (Republic of Slovenia, The Ministry of Agriculture, Forestry and Food, permission no. 34401-1/2015/7). B16.F10 murine melanoma cells in the exponential growth phase were used for the induction of subcutaneous tumors with the injection of 1 × 10^6^ cells in 100 µL of saline solution into the shaved right flank of mice. Tumors were allowed to grow approximately 4 days to reach a largest diameter of 3 mm before GET was performed (day 0). 

### 4.4. GET in Vitro

B16.F10 cells grown as a monolayer were collected in ice-cold electroporation buffer (125 mM sucrose, 10 mM K_2_HPO_4_, 2.5 mM KH_2_PO_4_, 2 mM MgCl_2_×6H_2_O). A dense cell suspension was prepared with a concentration of 25 × 10^6^ cells/mL. Plasmid DNA (11 µL) was added to 44 µL of prepared cell suspension and 50 µL of the resulting mixture (1 × 10^6^ cells and 10 µg of plasmid) were electroporated with an electric pulse generator (GT-01, Faculty of Electrical Engineering, University of Ljubljana, Ljubljana, Slovenia). Eight square-wave electric pulses with a voltage-to-distance ratio of 600 V/cm, pulse duration of 5 ms and frequency 1 Hz were delivered through two parallel stainless-steel plate electrodes with a 2 mm gap in between. After the application of electric pulses, the cells were incubated for 5 min with 100 µL of FBS and then plated in AMEM culture medium for further assays. Experimental groups were: addition of H_2_O alone (Control) or combined with application of electroporation (EP), addition of control plasmids (pControl, pVAX, pENTR/U6 scr) and GET of control plasmids (pControl + EP, pENTR/U6 scr + EP, pVAX + EP).

### 4.5. GET in Vivo

In vivo GET of control plasmids was performed on days 0 and 2 after the intratumoral (i.t.) injection of 100 µg plasmid DNA in 25 µL of endotoxin-free water. To ensure good conductivity in the contact of the electrodes with the skin, water based gel was applied (Ultragel, Budapest, Hungary). Ten minutes after plasmid injection, two sets of 4 pulses in perpendicular directions at a frequency of 1 Hz, amplitude over distance ratio 600 V/cm and 5 ms duration time were delivered through two parallel stainless-steel electrodes with a 4 or 6 mm distance between them. The pulses were generated by an electric pulse generator (ELECTRO cell B10, Betatech, Saint-Orens-de-Gameville, France). The animals were kept under inhalation anesthesia with 1.5% isoflurane (Izofluran Torrex para 250 mL, Chiesi Slovenia, Ljubljana, Slovenia) and an oxygen flow of 1 l/min during the entire procedure.

Experimental groups were as follows: Control (i.t. injection of endotoxin-free water), EP (i.t. injection of endotoxin-free water followed by application of electric pulses), pControl + EP (i.t. injection of pControl plasmid followed by application of electric pulses), pENTR/U6 scr + EP (i.t. injection of pENTR/U6 scr plasmid followed by application of electric pulses), pVAX + EP (i.t. injection of pVAX plasmid followed by application of electric pulses).

### 4.6. Tumor Growth

Tumor growth was followed after GET to quantify antitumor effects. Tumors were measured every second day after the first treatment in three orthogonal diameters with a Vernier caliper. Tumor volumes were calculated using the equation V = a × b × c × π/6. Tumor growth was monitored until the tumors reached approximately 350 mm^3^, thereafter the animals were euthanized. The response of tumors in mice that remained tumor-free for 100 days was termed as complete response. Tumor growth delay was determined as the difference in time when treated and untreated tumors reach a tumor volume of 40 mm^3^. The mice with complete response were excluded from the tumor growth delay calculation. 

### 4.7. Histology

Three days after the second delivery, three mice from each experimental group were sacrificed and tumors were excised and fixed in IHC zinc fixative (BD Pharmingen, BD Biosciences, San Jose, CA, USA) overnight and afterwards embedded in paraffin. Three consecutive 5-μm thick sections were cut from each paraffin block. The first section of each tumor sample was stained with hematoxylin and eosin (HE) for the determination of necrosis. The two remaining sections were immunohistochemically stained with rabbit monoclonal antibodies against Ki-67 (clone SP6, Thermo Fisher Scientific) at dilution 1:1200 for determination of proliferation of tumor cells or rabbit monoclonal antibodies against Cleaved Caspase-3 (Casp-3, Cell Signalling Technology, Danvers, MA, USA) at dilution 1:1500 for determination of apoptosis. As the colorogenic reagent a peroxidase-conjugated streptavidin–biotin system (Rabbit specific HRP/DAB detection IHC kit ab64261, Abcam, Cambridge, UK) was used followed by hematoxylin counterstaining. Three different fields of immunohistochemically stained sections were captured with a DP72 CCD camera (Olympus, Hamburg, Germany) connected to a BX-51 microscope (Olympus). Three independent observers blinded to the treatment groups quantified the percentage of tumor necrosis and the number of Ki-67- or Casp-3-positive cells on the acquired images.

### 4.8. Extraction of RNA from Cells or Tumors and Determination of DNA Sensors mRNAs by Quantitative Real-Time PCR (qPCR)

Four hours after GET in vitro, B16F.10 cells were collected to a cell suspension and centrifuged. Four hours after GET in vivo, mice were sacrificed and 3–6 tumors per experimental group were collected, snap frozen in liquid nitrogen, and homogenized in a mortar with a pestle. Total RNA was extracted from the samples (TRIzol Plus RNA Purification Kit, Thermo Fisher Scientific) according to the manufacturer’s instructions. The concentration and purity of extracted RNA was determined spectrophotometrically (Epoch, Winooski, VT, USA) by measurements of absorbance at 260 nm and of the ratio of absorbance at 260 and 280 nm, respectively. One µg of total RNA of appropriate purity (260/280 nm ratio = 1.95–2.05) was reverse transcribed into cDNA using SuperScript VILOTM cDNA Synthesis Kit (Thermo Fisher Scientific), according to the manufacturer’s instructions. Ten-fold cDNA dilutions were used for qPCR using SYBR Select Master Mix (Thermo Fisher Scientific) and custom primers for DDX60, DAI/ZBP1, p204, IFN1β and TNFα (Integrated DNA Technologies, Coralville, IA, USA) [17]. Quantitative PCR was performed on QuantStudio 3 Real-Time PCR System (Thermo Fisher Scientific). Relative quantification was performed by comparison to the housekeeping genes β-actin and glyceraldehyde 3-phosphate dehydrogenase using the ΔΔCt method [41]. 

### 4.9. Cytotoxicity Assay

After GET in vitro, B16.F10 cells from each experimental group were plated in 0.1 mL of cell culture medium in 96-well plates (Corning Incorporated, Corning, NY, USA). For groups without EP, 1000 cells were plated in each well and for groups with EP, 2000 cells were plated in each well. Cells were incubated at 37 °C in a 5% CO_2_ humidified incubator for 3 days. After incubation, Presto Blue^®^ viability reagent (Thermo Fisher Scientific) in medium was added to the wells and one hour later fluorescence intensity was measured with a microplate reader (Infinite 200, Tecan, Männedorf, Switzerland). The survival of cells for groups without EP was normalized to the control group and for groups with EP to EP group.

### 4.10. Giemsa Staining

Giemsa staining was performed 20 h after GET of control plasmids in order to observe morphological changes after GET of control plasmids. For this purpose, cytospins were prepared for each experimental group as follows: a funnel, labeled microscopy slide, and filter paper were fitted together and 1 × 10^3^ B16.F10 cells in 80 µL of PBS were added into the funnel. The samples were centrifuged at 1000 rpm for 4 min in cytocentrifuge (Cytospin 2, Thermo Shandon, Runcorn, UK). Slides were then air dried, fixed in methanol and stained with Giemsa’s Azure methylene blue solution (Merck, Darmstadt, Germany) according to the manufacturer’s protocol. Images were captured with a DP72 CCD camera connected to a BX-51 microscope.

### 4.11. Determination of Cell Death Mechanism

Cell death mechanisms of B16.F10 cells were determined with a FITC Annexin V Apoptosis Detection Kit with 7-AAD (BioLegend, San Diego, CA, USA) according to manufacturer’s instructions 2, 6 and 20 hours after GET of control plasmids. The measurements were performed with FACSCanto II flow cytometer (BD Biosciences, San Jose, CA, USA). 488-nm laser (air-cooled, 20 mW solid state) was used for the excitation and both 530 and 650-nm band-pass filter were used for detection of green and red fluorescence. For each cell sample, at least 40000 events were measured. The data were analyzed by the BD FACSDiva software version 8.0.1 (BD Biosciences, San Jose, CA, USA). All debris was excluded for further analysis by using FSC/SSC scatter plot. The experiments were performed twice in 3 parallels. 

### 4.12. Statistical Analysis

Data were first tested for normality of distribution with the Shapiro-Wilk test and were represented as arithmetic means (AM) with error bars representing standard errors (SEM). The difference among the groups was evaluated by one-way analysis of variance followed by a Holm-Sidak test for multiple comparisons to determine the significance among all group or Holm-Sidak test for comparison to control only (no EP) or EP group. All groups were compared with each other. A *p*-value of less than 0.05 was considered to be statistically significant. Linear correlation between the results of the two methods was evaluated by the Pearson’s correlation coefficient. SigmaPlot Software (Systat Software, Chicago, IL, USA) was used for statistical analysis and graphical representation.

## 5. Conclusions 

In summary, although differences in antitumor effects were observed among control plasmids in vivo, no differences in cellular responses to plasmid GET were detected in tumor cells in vitro. Other cells present in the tumor microenvironment, including fibroblasts, endothelial cells, and particularly immune cells may be responsible for the effects. Furthermore, also the properties related to plasmids contribute to the observed antitumor effectiveness.

## Figures and Tables

**Table 1:** Calculated tumor growth delay and complete response rate after GET of control plasmids.

Group	N	Growth Delay (to 40 mm^3^) (days) (AM ± SEM)	Complete Response
Control	12	0.0 ± 0.4	0
EP	12	0.7 ± 0.1	0
pControl + EP	12	4.6 ± 1.5 *	1/12 (8.3%)
pENTR/U6 scr + EP	12	7.4 ± 0.6 *	3/12 (25%)
pVAX + EP	6	10.5 ± 2.4 *^,^**	2/6 (33%)

AM, arithmetic mean; SEM, standard error of the mean; N = number of parallels in two independent experiments; * *p* < 0.05 statistically significant difference compared to control and EP groups; ** *p* < 0.05 statistically significant difference compared to pControl + EP group.

**Table 2:** Expression of mRNA for DNA sensors (DDX60, DAI/ZBP1, p204) and cytokines (IFN β and TNF α) in B16.F10 tumors after GET of control plasmids.

Group	N	DNA Sensors			Cytokines	
		DDX60 (AM ± SEM)	DAI/ZBP1 (AM ± SEM)	p204 (AM ± SEM)	IFN β (AM ± SEM)	TNF α (AM ± SEM)
Control	3	1.09 ± 0.31	1.06 ± 0.26	1.22 ± 0.57	1.13 ± 0.42	1.06 ± 0.26
EP	3	1.05 ± 0.15	0.98 ± 0.18	1.63 ± 0.19	13.27 ± 3.36	0.61 ± 0.12
pContol + EP	6	2.41 ± 0.23	2.43 ± 0.33	3.57 ± 0.61	126.22 ± 20.03 *	9.98 ± 1.63
pENTR/U6 scr + EP	6	39.42 ± 20.86	14.16 ± 5.25	15.62 ± 4.49 **	967.45 ± 184.35 *	5.08 ± 2.47
pVAX + EP	6	5.21 ± 1.45	5.88 ± 2.55	11.39 ± 2.37	743.48 ± 174.34 *	12.08 ± 2.60 *

AM, arithmetic mean; SEM, standard error of the mean; N = number of parallels in two independent experiments; * *p* < 0.05 statistically significant difference compared to control and EP groups. ** *p* < 0.05 statistically significant difference compared to control, EP and pControl + EP groups, respectively; AM was calculated as the average of 2^−ΔΔCt^ values.

**Table 3:** Expression of mRNA for cytosolic DNA sensors and pathway activation markers after GET of three different control plasmids in B16.F10 melanoma cells.

Group	N	DNA Sensors			Cytokines	
		DDX60 (AM ± SEM)	DAI/ZBP1 (AM ± SEM)	p204 (AM ± SEM)	IFN β (AM ± SEM)	TNF α (AM ± SEM)
Control	8	1.00 ± 0.04	1.01 ± 0.06	1.01 ± 0.04	1.01 ± 0.05	1.28 ± 0.29
pContol + EP	8	39.30 ± 5.52 *	109.85 ± 24.74	64.83 ± 16.46 *	74.43 ± 27.54	1.69 ± 0.43
pENTR/U6 scr + EP	8	52.74 ± 14.01 *	161.78 ± 54.23 *	68.76 ± 21.38 *	78.90 ± 19.86	2.44 ± 0.98
pVAX + EP	8	62.56 ± 13.29 *	153.85 ± 60.12 *	82.25 ± 28.97 *	102.88 ± 27.46 *	4.35 ± 1.81

AM, arithmetic mean; SEM, standard error of the mean; N = number of parallels in two independent experiments; * *p* < 0.05 statistically significant difference compared to control group; AM was calculated as the average of 2^−ΔΔCt^ values.

**Table 4:** Comparative analysis of control plasmids’ characteristic, which may potentially contribute to antitumor effectiveness after in vivo GET.

Plasmid	Base Pairs	Molecular Weight *	Copies/100 ug	Motifs **/Copy	Total Motifs ** Injected	Promotor	Antibiotic Resistance Gene
pVAX1	2999	1.85 × 10^6^	3.25 × 10^13^	7	2.27 × 10^14^	CMV	kanamycin
pControl	3390	2.09 × 10^6^	2.87 × 10^13^	10	2.87 × 10^14^	CMV	ampicillin
pENTR/U6 scr	2909	1.79 × 10^6^	3.35 × 10^13^	13	4.36 × 10^14^	U6	kanamycin

* with precise sequence, http://scienceprimer.com/nucleotide-molecular-weight-calculator; ** Calculation of number of mouse specific CpG motifs injected into tumors (100 ug DNA) per Yu et al., 2003; irrespective of relative immunostimulatory activity [40].
